# A Co-Culture System for Studying Cellular Interactions in Vascular Disease

**DOI:** 10.3390/bioengineering11111090

**Published:** 2024-10-30

**Authors:** Abirami M. Padmanaban, Kumar Ganesan, Kunka Mohanram Ramkumar

**Affiliations:** 1 Department of Biotechnology, School of Bioengineering, SRM Institute of Science and Technology, Kattankulathur 603 203, Tamil Nadu, India; abiramim3@srmist.edu.in; 2 School of Chinese Medicine, Li Ka Shing Faculty of Medicine, The University of Hong Kong, 10 Sassoon Road, Pokfulam, Hong Kong 999077, China; kumarg@hku.hk

**Keywords:** cardiovascular diseases, endothelial dysfunction, co-culture models, cellular interactions, vascular pathology

## Abstract

Cardiovascular diseases (CVDs) are leading causes of morbidity and mortality globally, characterized by complications such as heart failure, atherosclerosis, and coronary artery disease. The vascular endothelium, forming the inner lining of blood vessels, plays a pivotal role in maintaining vascular homeostasis. The dysfunction of endothelial cells contributes significantly to the progression of CVDs, particularly through impaired cellular communication and paracrine signaling with other cell types, such as smooth muscle cells and macrophages. In recent years, co-culture systems have emerged as advanced in vitro models for investigating these interactions and mimicking the pathological environment of CVDs. This review provides an in-depth analysis of co-culture models that explore endothelial cell dysfunction and the role of cellular interactions in the development of vascular diseases. It summarizes recent advancements in multicellular co-culture models, their physiological and therapeutic relevance, and the insights they provide into the molecular mechanisms underlying CVDs. Additionally, we evaluate the advantages and limitations of these models, offering perspectives on how they can be utilized for the development of novel therapeutic strategies and drug testing in cardiovascular research.

## 1. Introduction

Vascular diseases, including cardiovascular diseases (CVDs), represent a significant global health concern, comprising disorders that affect the heart and blood vessels [1]. Despite notable advancements in diagnosis and treatment over recent years, CVD is still the foremost cause of morbidity and mortality, leading to economic burden on health care systems. Vascular diseases like atherosclerosis and hypertension, and diabetes-related vascular complications, play a central role in the development of CVDs, leading to conditions such as heart attacks, strokes, and peripheral artery disease [2]. Atherosclerosis, a major contributor to vascular diseases, involves the accumulation of plaques within the arterial walls, which restricts blood flow and can lead to life-threatening events [3]. Endothelial dysfunction, inflammation, oxidative stress, and lipid buildup are central drivers of atherosclerosis, particularly exacerbated by chronic conditions such as hypertension and diabetes [4,5]. These conditions impair endothelial cells, increase oxidative stress, and promote inflammatory responses, accelerating vascular injury [6,7].

Given the complexity of vascular diseases, where several cell types (endothelial cells, immune cells, and smooth muscle cells) interact to mediate disease progression, there is an important necessity to understand the underlying cellular mechanisms. Tremendous efforts have been devoted to CVD research, employing both in vivo and in vitro models. However, human studies are limited by the scarcity of heart and vessel tissues, which are difficult to obtain for experimental purposes. Consequently, most research relies on in vitro culture techniques using human primary cells or in vivo rodent models. While rodent models give us insights into the pathophysiology of vascular diseases, they may not fully replicate the human condition.

To bridge this gap, co-culture systems have emerged as essential tools for simulating the complex interactions between various cell types involved in vascular diseases [8]. By mimicking the in vivo cellular environment, co-culture systems enable researchers to study how endothelial cells, immune cells, and smooth muscle cells communicate and contribute to vascular dysfunction. These systems provide a controlled platform for dissecting the molecular signaling cascades and identifying potential therapeutic targets, offering a more precise approach to understanding and treating vascular diseases.

In the present review, we aim to summarize the various co-culture systems and cellular interactions within cardiovascular co-culture models. We detail the common cell-to-cell interactions that contribute to vascular diseases, including those among endothelial cells, macrophages, smooth muscle cells, monocytes, and cardiomyocytes. Overall, this review provides insights into how cellular interactions are studied in different microenvironments and how these interactions contribute to the development of specific cardiovascular diseases.

### 1.1. Role of Cellular Interactions in Vascular Disease

Vascular diseases, including atherosclerosis and hypertension, and diabetic vascular complications, are complex conditions driven by the complex interplay between various cell types within blood vessels. Understanding how these cellular interactions contribute to the pathophysiology of vascular disease is crucial for developing targeted therapies. Key players in this dynamic environment include endothelial cells, cardiomyocytes, smooth muscle cells, immune cells, and fibroblasts, with each contributing distinct, but interconnected roles.

### 1.2. Endothelial Cells (ECs)

**Figure 1:**
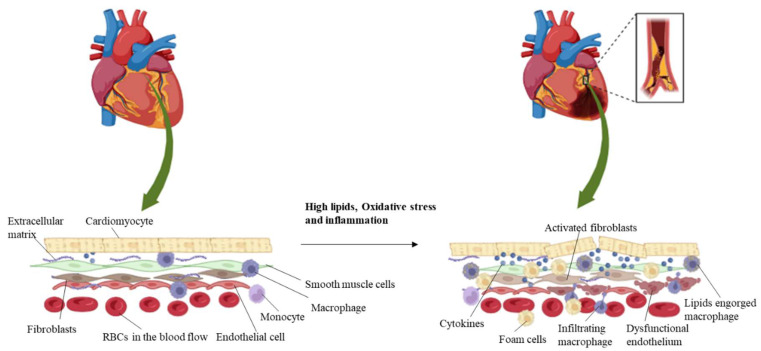
Schematic representation of the multi-cellular components of the myocardial environment. Atherosclerotic lesions consist of three important components: a fibrous part composed of extracellular lipids and connective tissue matrix, a cellular part composed of immune cells (monocytes, macrophages), and smooth muscle cells and foam cells, which are formed by the accumulation of lipids in macrophages. Cardiomyocytes associate with the neighboring cells such as endothelial cells and fibroblasts and aid in their cellular function. Immune cells adhere to the vascular tissue upon disease condition, and the macrophages engulf lipoproteins to form foam cells, which accelerates atherosclerosis.

At the innermost lining of the blood vessels, endothelial cells serve as a critical barrier between circulating blood and the vascular wall. In healthy conditions, ECs support vascular homeostasis by regulating blood flow, preventing clot formation, and controlling inflammatory responses [9,10]. However, in vascular disease, endothelial dysfunction is a key early event. Factors like oxidative stress, hyperlipidemia, and high blood pressure damage ECs, leading to increased permeability, impaired nitric oxide production, and the expression of adhesion molecules that attract immune cells [11,12]. Endothelial dysfunction promotes vascular inflammation and contributes to plaque formation in atherosclerosis (Figure 1) [13,14].

### 1.3. Smooth Muscle Cells (SMCs)

SMCs are found in the middle layer of blood vessels. They play a fundamental role in regulating vascular tone and blood pressure. In response to injury or inflammation, SMCs switch from a contractile phenotype to a synthetic one, proliferating and migrating to the inner layer of the vessel wall [15]. This phenotypic switch contributes to the thickening of the vessel wall and the formation of atherosclerotic plaques (Figure 1) [16]. SMCs also produce extracellular matrix components, leading to fibrosis and stiffening of the arteries, further exacerbating vascular disease [17,18].

### 1.4. Immune Cells

Immune cells, including macrophages, T-cells, and monocytes, are key mediators of vascular inflammation. In atherosclerosis, monocytes adhere to dysfunctional endothelial cells and migrate into the vessel wall, where they differentiate into macrophages [19]. These macrophages engulf oxidized lipids and transform into foam cells, which is a hallmark of atherosclerotic plaques (Figure 1). As foam cells accumulate, they release pro-inflammatory cytokines and reactive oxygen species, perpetuating inflammation and promoting the further recruitment of immune cells [20]. T-cells, particularly T-helper (Th1) cells, also contribute to inflammation by secreting cytokines like interferon-gamma (IFN-γ), which activates macrophages and enhances vascular damage [12,21].

### 1.5. Fibroblasts

Fibroblasts are involved in the repair and remodeling of vascular tissue following injury. However, their excessive activation in response to chronic inflammation or injury can lead to fibrosis, a characteristic feature of advanced vascular diseases. Activated fibroblasts produce large amounts of extracellular matrix proteins, such as collagen, leading to stiffening of the vessel wall and further impairing vascular function (Figure 1) [22]. In association with fibrosis, fibroblasts also interact with endothelial cells and immune cells, influencing the overall inflammatory milieu in vascular diseases.

### 1.6. Platelets

Though not residing in the vessel wall, platelets play a crucial role in vascular disease, especially in thrombosis and atherosclerosis [23,24]. Because platelets preserve blood vessel integrity, they are essential for haemostasis. They serve as the first line of defense in the event of an injury, developing thrombi that repair damaged endothelium tissue, and are essential for maintaining haemostasis [25].

### 1.7. Cardiomyocytes (CMs)

Cardiomyocytes (CMs) are large muscle cells of the cardiac tissue and are the most energetic cells in the system. During inflammation or injury, the paracrine signals from other cell types regulate cardiomyocytes [26].

### 1.8. Extracellular Matrix (ECM)

The ECM, produced mainly by SMCs and fibroblasts, is not merely a structural component of blood vessels, but also plays a critical role in cellular interactions. The composition and integrity of the ECM are altered in vascular diseases, affecting how cells respond to mechanical and biochemical signals [27]. Degradation of the ECM by enzymes such as matrix metalloproteinases (MMPs), secreted by macrophages and SMCs, can destabilize atherosclerotic plaques, making them more prone to rupture and leading to heart attacks and strokes [28,29].

### 1.9. Pericytes

Pericytes are specialized contractile cells located on the abluminal surface of endothelial cells in capillaries and microvessels, playing essential roles in vascular biology. They are crucial for maintaining vascular stability and integrity by regulating blood flow, supporting endothelial cell function, and contributing to the formation and remodeling of blood vessels during development and wound healing [30]. Pericytes also participate in angiogenesis, responding to growth factors and cytokines released from surrounding cells, and they can differentiate into various cell types, such as smooth muscle cells and fibroblasts, depending on the microenvironment [31]. Furthermore, they play a protective role in the blood–brain barrier and are involved in the pathophysiology of various diseases, including diabetes and cancer, where their dysfunction can lead to vascular complications and impaired tissue repair [32].

## 2. Interaction Dynamics in Vascular Disease

### 2.1. Endothelial–Smooth Muscle Cell Interaction

Endothelial cells and SMCs communicate through paracrine signaling. In healthy vessels, ECs release nitric oxide (NO) to inhibit SMC proliferation and maintain vessel tone [33]. In vascular disease, endothelial dysfunction reduces NO production, leading to SMC migration and proliferation, and contributing to plaque formation and vessel narrowing [34]. In conditions such as atherosclerosis, the interaction between ECs and SMCs is altered, leading to endothelial dysfunction and enhanced SMC proliferation and migration [35]. This pathological crosstalk contributes to plaque formation and vascular remodeling. Studies have shown that exposing SMCs to inflammatory cytokines can induce changes in ECs, promoting a pro-inflammatory state that accelerates vascular disease progression [36].

Endothelial cells and smooth muscle cells play central roles in preserving vascular health and function through their interactions, which regulate vascular tone, inflammation, and remodeling. In healthy blood vessels, ECs form a monolayer lining that acts as a barrier and regulates vascular permeability, while SMCs, located in the vessel wall, contribute to vascular tone by contracting and relaxing in response to various stimuli [37,38]. The inter-communication between these two critical cell types is crucial for sustaining homeostasis within the vascular system. Co-culture studies have provided significant insights into the pathophysiological mechanisms underlying vascular diseases such as atherosclerosis and restenosis [39,40]. For instance, the release of signaling molecules like nitric oxide (NO) from ECs can induce the relaxation of adjacent SMCs, thereby regulating blood flow and pressure. Conversely, pro-inflammatory cytokines produced by SMCs can alter the function of ECs, promoting a pro-inflammatory environment that is conducive to atherosclerosis [41]. In co-culture models that simulate atherosclerotic conditions, researchers have observed that the interaction between ECs and SMCs leads to the enhanced expression of adhesion molecules on ECs, facilitating the recruitment of inflammatory cells such as monocytes and macrophages [42]. This recruitment contributes to the formation of atherosclerotic plaques, highlighting the critical role of EC–SMC interactions in disease progression [43]. Moreover, studies have shown that co-culturing SMCs with ECs in a 3D environment can lead to changes in the expression of extracellular matrix components, which are essential for vascular remodeling and repair processes [44]. When endothelial cells and smooth muscle cells were exposed to laminar pulsatile and disturbed flow, a defective endothelial layer was found, which could lead to intimal hyperplasia by the accumulation of smooth muscle cells [45].

Additionally, research into restenosis, characterized by the re-narrowing of blood vessels following intervention, has benefited from co-culture studies. These investigations reveal how SMC proliferation and migration, stimulated by growth factors released from ECs, contribute to neointimal hyperplasia, a hallmark of restenosis. By understanding the dynamics of EC–SMC interactions in various states of health and disease, researchers are better positioned to develop therapeutic strategies aimed at mitigating vascular pathologies and improving patient outcomes [46]. The infiltration of lipids into macrophages and development of foam cell was studied in a co-culture of endothelial cells, monocytes, and smooth muscle cells in a scaffold [47]. A three-cell co-culture model of atherosclerosis was established by culturing ECs and SMCs, along with macrophages, and an increase in cytokines and inflammation was determined (Table 1) [48].

### 2.2. Endothelial–Immune Cell Interaction

Damaged endothelial cells show adhesion molecules (e.g., VCAM-1 and ICAM-1) that mediate immune cell recruitment. The interaction between endothelial cells and immune cells, especially macrophages, creates a pro-inflammatory environment, promoting plaque progression in atherosclerosis [57]. Under normal physiological conditions, ECs serve as a barrier, regulating the passage of immune cells into tissues [58]. However, during pathological states, such as atherosclerosis, ECs become activated and express adhesion molecules (e.g., VCAM-1 and ICAM-1), which promote the adhesion and migration of circulating immune cells into the vascular wall [59,60]. Co-culturing macrophages with ECs has shown that activated macrophages secrete pro-inflammatory cytokines, such as TNF-α and IL-6, which further stimulate endothelial activation and permeability [61]. This reciprocal signaling amplifies the inflammatory response and contributes to plaque formation and instability.

Endothelial cells (ECs) serve as the front line of defense in the vascular system, actively engaging with immune cells such as monocytes, macrophages, and T-cells to regulate vascular inflammation and plaque formation. Under physiological conditions, ECs maintain vascular homeostasis and promote immune tolerance. However, in the context of vascular diseases like atherosclerosis, the interactions between ECs and immune cells become dysregulated, contributing to inflammation and plaque development [57,58]. Macrophages play a central role in the initiation and development of atherosclerosis. In response to endothelial injury or dysfunction, activated ECs could express adhesion molecules and chemokines that promote the attachment of circulating monocytes, which eventually differentiate into macrophages upon entering the vessel wall. Once within the intima, macrophages engulf oxidized low-density lipoprotein (LDL) particles, transforming into foam cells, a key component of atherosclerotic plaques [62].

Co-culture systems that simulate EC–immune cell interactions have provided valuable insights into this crosstalk. For instance, studies have demonstrated that co-culturing ECs with macrophages results in the increased secretion of pro-inflammatory cytokines, such as tumor necrosis factor-alpha (TNF-α) and interleukin-6 (IL-6), which further enhances endothelial permeability and promotes leukocyte adhesion [50]. This inflammatory cascade fosters a vicious cycle of endothelial dysfunction and immune activation, exacerbating plaque formation. Another study assessing the role of multicellular interactions in vascular angiogenesis, found that the interactions were determined by establishing the angiotrophic stimulation by co-culturing endothelial cells and macrophages. Macrophages enhanced angiogenesis via increasing the length and number of endothelial sprouts. Authors have also analyzed the impact of pericytes by co-culturing with endothelial cells and found an additive effect on angiogenesis in the vasculature [51].

Additionally, the interaction between ECs and T-cells has been determined to influence vascular inflammation. Co-culture studies indicate that activated T-cells can alter endothelial cell behavior, promoting a pro-inflammatory state characterized by the expression of major histocompatibility complex (MHC) molecules and co-stimulatory ligands [63]. This interaction not only enhances T-cell activation and proliferation, but also facilitates the movement of other immune cells to the inflammatory site, further contributing to plaque instability. Activated macrophages releases factors that contribute to endothelial dysfunction. A study determined the role of peroxynitrite-induced, macrophage-mediated endothelial cell injury in hypoxia by the co-culture of macrophages and human cardiac microvascular endothelial cells (HCMECs). Results suggested an increase in hypoxic proteins and prostaglandins, revealing that peroxynitrite contributes to endothelial injury in the co-culture [49]. A co-culture experiment of endothelial cells and macrophages with exposure to oxidized LDL demonstrated that endothelin-1 (ET-1) can affect the macrophages and promote migration and M1 macrophage activation [50].

Remarkably, a study investigated the mechanism of hyperglycemic arteriosclerosis by evaluating the effects of advanced glycation end products (AGEs) on cytokine synthesis and smooth muscle cell proliferation via co-culturing aortic vascular smooth muscle cells (SMCs), monocytes (THP1), and endothelial cells (HUVECs). These complex interactions revealed that co-culture is a valuable tool to determine vascular cell–cell interactions under the influence of AGEs (Table 1) [52].

### 2.3. Endothelial–Fibroblast Interactions

Endothelial cells (ECs) and fibroblasts play crucial roles in vascular health and disease, particularly in the context of fibrosis and vascular stiffness associated with conditions such as hypertension. The interaction between these two different cell types is essential for maintaining vascular integrity and function, yet dysregulation can lead to pathological changes that contribute to cardiovascular complications.

In the setting of hypertension, chronic pressure overload induces mechanical stress on the endothelium, triggering a cascade of cellular responses. ECs respond to this stress by releasing various cytokines and growth factors, including transforming growth factor-beta (TGF-β) and platelet-derived growth factor (PDGF) [64]. These factors are pivotal in promoting fibroblast activation and recruitment, leading to the increased accumulation of extracellular matrix (ECM) components and, ultimately, vascular fibrosis.

Fibroblasts, upon activation by pro-inflammatory cytokines released from ECs, undergo a phenotypic transformation into myofibroblasts, characterized by increased contractility and ECM production [65]. This process contributes to vascular stiffness and impaired vascular compliance, which are hallmarks of hypertensive vascular disease. The excessive deposition of ECM proteins, such as collagen and elastin, alters the structural integrity of the blood vessel wall, promoting rigidity and diminishing its ability to respond to hemodynamic changes [66]. Studies have shown that co-culturing ECs with fibroblasts enhances the secretion of ECM proteins, indicating a synergistic effect on fibrosis [67]. Additionally, these experiments demonstrate that activated fibroblasts can influence endothelial function by promoting EC permeability and inflammation, creating a feedback loop that exacerbates vascular remodeling [68].

Moreover, co-culture systems have been used to assess the effect of specific signaling pathways on the interactions between ECs and fibroblasts. For example, the inhibition of the TGF-β signaling pathway in co-cultured cells has been shown to reduce myofibroblast differentiation and ECM deposition, highlighting potential therapeutic targets for mitigating vascular stiffness and fibrosis [69].

When human dermal fibroblasts were co-cultured with HUVECs on a bioceramic, the paracrine effect of these cells induced angiogenesis via facilitating tube formation [70]. Another interesting study revealed that the co-culture of fibroblasts with HUVECs improved the maturation of microvessels in fibrin polymer filaments compared to the addition of VEGF and bFGF. This study demonstrates that fibroblasts stimulate angiogenesis and promote the migration of endothelial cells through co-culture experiments [71]. The precise role of cardiac fibroblasts in initiating the proliferation, maturation, and formation of vessel sprouts by ECs was determined by co-culturing them. It was discovered that fibroblasts offered a good support system for angiogenic sprout formation of endothelial cells when compared with mesenchymal stem cells [72].

An additional study established that fibroblasts support the formation of endothelial cell tubular structures in a 3D spheroid co-culture model upon culturing human microvascular endothelial cells and fibroblasts [73,74]. Human cardiac microvascular endothelial cells (HCMVECs) treated with doxorubicin when co-cultured with cardiac fibroblasts determined the suppression of vascular network formation, which shows drug-related cardiomyopathy development [64].

### 2.4. Smooth Muscle Cell–Immune Cell Interaction

Immune cells, particularly macrophages, secrete cytokines and growth factors that influence SMC behavior. These signals stimulate SMC proliferation and ECM production, contributing to vascular remodeling and stiffening. Under normal conditions, SMCs contribute to vascular tone and homeostasis. However, during injury or disease, they undergo phenotypic modulation, transitioning from a contractile to a synthetic phenotype, which is characterized by the increased proliferation and secretion of extracellular matrix components [75,76]. Activated macrophages release pro-inflammatory cytokines and growth factors (e.g., IL-1β, IL-6, and PDGF) that can promote SMC proliferation and migration, leading to neointimal hyperplasia and vascular remodeling [77,78]. This interaction is specifically important in the pathology of restenosis, where SMC activation and immune cell recruitment contribute to excessive tissue growth following vascular injury. Additionally, SMCs can also modulate immune cell function. SMCs have been shown to express immune-modulatory molecules, such as programmed death-ligand 1 (PD-L1), which can inhibit T-cell activation and promote an immunosuppressive environment [79].

### 2.5. Immune Cell–Fibroblast Interaction

The interplay between immune cells and fibroblasts is a principal component in the pathogenesis of various vascular diseases, particularly those involving fibrosis and tissue remodeling. Fibroblasts, the primary effector cells in connective tissue, play a central role in preserving the structural integrity of the vascular system and responding to injury. When tissue is damaged, immune cells, such as macrophages and T-cells, infiltrate the site and secrete a variety of cytokines and growth factors that influence fibroblast behavior [80]. Macrophages release factors like TGF-β, which is known to drive fibroblast activation and the transition to a myofibroblast phenotype, a key player in tissue repair and fibrosis [81]. This process can be detrimental in vascular diseases, as excessive fibroblast activation leads to vascular stiffness and impaired functionality. Conversely, fibroblasts also modulate immune responses. They can produce immunomodulatory cytokines that influence the activation and polarization of immune cells, thus shaping the local immune environment [82,83]. Fibroblasts can secrete IL-6, which can promote the differentiation of naive T-cells into pro-inflammatory Th17 cells [84]. Understanding the dynamics of immune cell–fibroblast interactions through co-culture systems provides valuable insights into the mechanisms driving vascular disease progression.

### 2.6. Endothelial Cell and Cardiomyocyte Interactions

Every cardiomyocyte is in physical contact with at least one endothelial cell, and this arrangement makes way for paracrine and mechanical crosstalk in between the ECs and CMs. This cellular interaction between ECs and CMs plays an important role in the development and regulation of CM function [85,86]. The co-culture of endothelial and cardiomyocytes has determined that the cellular interaction increases the susceptibility of CMs to ischemia-reperfusion injury [56]. In a diabetic study of atherosclerosis, mouse cardiac endothelial cells and cardiomyocytes from Goto-Kakizaki rats were co-cultured, and the results showed that cardiomyocytes mediated an anti-angiogenic effect in hyperglycemic conditions through the exosomal transfer of miR-320 to the endothelial cells [53]. A recent study of co-culturing endothelial cells and cardiomyocytes demonstrated a protective role of curcumin, since it inhibited the cardiomyocyte cell death in hypoxic conditions. Similarly, a co-culture study of endothelial cells overexpressing rhSLPI (secretory leukocyte protease inhibitor) and cardiomyocytes revealed a cardioprotective effect of SLPI (Table 1) [55].

### 2.7. Endothelial Cell–Platelet Interactions

Activated platelets adhere to damaged endothelial cells and release growth factors and chemokines that recruit immune cells and promote smooth muscle cell migration [87]. Platelet–endothelial and platelet–leukocyte interactions further amplify inflammation and contribute to plaque development and thrombosis [88]. Indeed, a study examined the effect of platelet migration toward endothelial cells by co-culturing endothelial cells overlaid with monocytes and platelets in a trans-well insert. The soluble factors secreted by the monocytes facilitated the migration of platelets to the activated endothelium, effectively replicating the interactions of platelets and monocytes during atherogenesis [89].

### 2.8. Endothelial Cell–Pericyte Interaction

In order to sustain angiogenic sprouts and promote the differentiation of ECs, pericytes are recruited by ECs through factors like PDGF-BB, playing a critical role in blood vessel growth [31]. The disruption of pericyte–EC connections has been associated with a variety of disease pathologies and can lead to altered vascular function. Co-culture studies have indicated that pericytes can influence microvascular endothelial cells without direct contact, primarily by secreting soluble mediators. It has been shown that exposure to pericyte-conditioned media significantly increases the mRNA expression of the potent vasoconstrictor endothelin-1 in endothelial cells [90]. Subsequent studies further demonstrated that pericytes release factors into the conditioned media that regulate endothelial cell migration and tubule formation [91].

## 3. Importance of In Vitro Co-Culture Systems

In vitro co-culture systems are a powerful tool for studying intricate cellular interactions, particularly in the context of vascular diseases. Traditional monoculture models, where a single cell type is studied in isolation, fail to capture the dynamic interactions between different cell types that are critical to disease progression. Co-culture systems, on the other hand, allow for the simultaneous study of multiple cell types in a controlled environment, offering several key advantages.

### 3.1. Mimicking Physiological Conditions

Vascular diseases, such as atherosclerosis, involve the coordinated interplay of fibroblasts, smooth muscle cells, immune cells, and endothelial cells. Co-culture systems enable the recreation of these interactions, providing a more physiologically relevant model to study disease mechanisms [92]. By replicating the multicellular environment of blood vessels, researchers can better understand how different cell types communicate and influence each other during disease progression [93].

### 3.2. Studying Cell–Cell Communication

In vascular diseases, cellular communication through direct contact, soluble factors, and extracellular matrix components plays a critical role. Co-culture systems allow for the investigation of these cell–cell interactions in real time [93]. For example, endothelial cells may release factors that regulate smooth muscle cell migration, while immune cells can alter the behavior of both endothelial and smooth muscle cells through inflammatory signaling [94,95].

### 3.3. Modeling Disease Pathogenesis

Co-culture systems offer a precise representation of the cellular microenvironment in diseased vessels. For example, in atherosclerosis, interactions between endothelial cells and immune cells (such as macrophages) can be studied to understand how inflammation drives plaque formation [96,97,98]. Similarly, co-cultures of smooth muscle cells and fibroblasts can be used to investigate vascular remodeling and fibrosis, both key aspects of vascular pathology [99].

### 3.4. Testing Drug Efficacy

The effectiveness of therapeutic agents often depends on how they affect multiple cell types in the disease microenvironment. Co-culture models provide a more realistic platform for drug testing by allowing researchers to evaluate the impact of a drug on several cell types simultaneously [100,101]. This can lead to better predictions of drug efficacy and toxicity in complex tissues like blood vessels.

### 3.5. Uncovering Novel Therapeutic Targets

By studying interactions between different cell types, co-culture systems can reveal novel therapeutic targets that might be overlooked in single-cell studies. For instance, the crosstalk between endothelial cells and immune cells could identify new pathways involved in inflammation, while interactions between smooth muscle cells and fibroblasts might uncover novel regulators of vascular remodeling [96,97,99].

### 3.6. Personalized Medicine and Tissue Engineering

Co-culture systems can be customized to include patient-derived cells, providing a platform for personalized medicine. Researchers can study how different individuals’ cells interact in disease contexts and test personalized treatments [102,103]. Additionally, co-culture models are vital for tissue engineering, where the goal is to create functional blood vessels or vascularized tissues for transplantation or therapeutic applications.

## 4. Types of Co-Culture Systems

Co-culture systems are invaluable components for studying interactions between different cell types, offering a practical method to introduce a desired stimulus from one cell type to another. This approach leverages the natural crosstalk that occurs between cells, either through direct cell–cell contact or soluble factors, mimicking physiological interactions observed in tissues during regeneration, wound healing, and development [104,105]. This technique is often more cost-effective than adding specific growth factors or activators into the culture medium, as it can replicate similar biological effects.

Co-culture models are particularly relevant to drug development and disease modeling, as they provide an in vivo-like environment. Understanding the mechanisms of cellular crosstalk in these systems is essential for investigating disease pathways and therapeutic interventions [93]. Moreover, certain cell types may not grow effectively in monoculture, or they may not display the physiological characteristics seen in vivo, yet they can thrive and exhibit desired behaviors when grown in co-culture systems [106]. Co-culture systems are frequently employed to study the interactions between various cell types, such as fibroblasts, adipocytes, endothelial cells, macrophages, and smooth muscle cells. These systems are critical for unraveling the metabolic and cellular interactions between tissues, such as adipose and vascular tissues [107]. Co-culture studies offer valuable insights into how cells communicate through secreted factors, which can impact metabolic functions like energy homeostasis, oxidative stress, and inflammation. For example, a study investigating the co-culture relationship between macrophages and adipocytes explored how these cells interact under conditions mimicking obesity, insulin resistance, and inflammation, highlighting the importance of cellular interactions in metabolic diseases [108].

Co-culture models can effectively mimic the spatial organization of different cell types within a wound environment. Co-culture models can simulate both proximal and distal arrangements of cells, allowing us to replicate how different cell types interact based on their physical proximity in the wound [109,110]. Layering one cell type above another can mimic the vascular structure, reflecting the degree of cell location in the wound. This method is particularly useful in modeling the complex architecture of tissues where endothelial cells, fibroblasts, and other cell types are layered [111]. Co-culture systems can incorporate shear stress, which influences cellular interactions by simulating the dynamic environment of blood flow, further enhancing the physiological relevance of the model [112]. Factors such as hyperglycemia, hyperlipidemia, hypoxia, and nutrient availability significantly affect cellular interactions [113]. By adjusting these conditions in co-culture models, we can better mimic pathological states such as diabetic wounds, where cellular behavior and communication are altered.

Below are the key types of co-culture systems used in vascular research.

### 4.1. Direct Co-Culture

**Figure 2:**
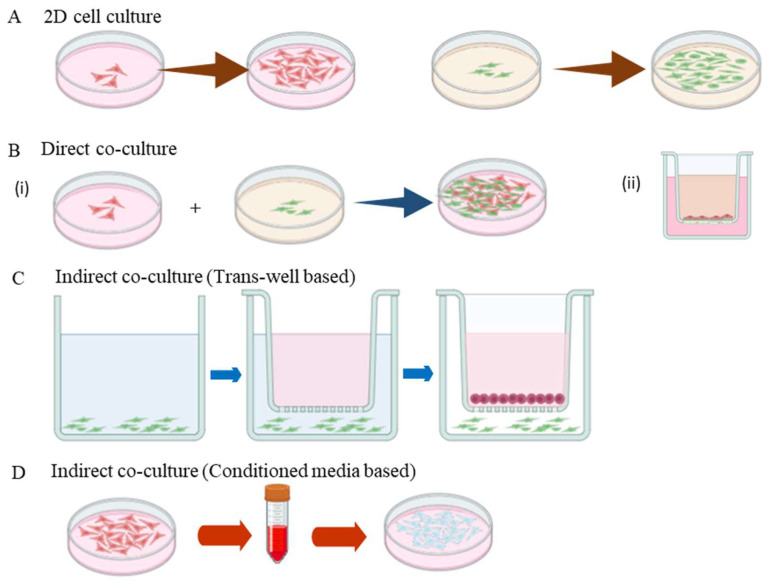
Illustration of the different types of 2D cell culture. (**A**) Formation of monolayer by a single type of cell in the Petri dish. (**B**) Direct contact co-culture model: (**i**) Two different types of cells are mixed at a standard ratio and inoculated on the same culture dish, and (**ii**) a trans-well-based model where each cell type is cultured to the opposite sides of the permeable membrane in the trans-well chamber. This type of culture ensures physical contact and juxtacrine and paracrine signaling, and these cells tend to maintain their function and structure. (**C**) Indirect co-culture model with trans-well insert ensure the cell–cell interactions mediated by secreted factors are released in the culture media. (**D**) Conditioned media-based co-culture system, where the cellular secretions from one cell type are transferred to the other cell type.

When different types of cells are grown together under same environment, allowing physical contact between the cells is referred to as direct co-culture. This interaction is mediated through proteins present on the cell surface, which closely mimics in vivo conditions. Direct co-culture enhances signal transduction between different cell types through this direct contact [114]. These co-culture models can be scaffold-based or non-scaffold-based, with results potentially varying based on the nature of the scaffold used and the ratio of the two cell types seeded. In this setup, juxtacrine signaling can also occur, where signaling molecules or proteins from one cell type interact with receptors on an adjacent cell type. Therefore, a combination of secreted factors, cell–cell communication, physical contact, and both paracrine and juxtacrine signaling are the driving features of the direct co-culture system (Figure 2) [115]. Interactions in a direct co-culture occur through gap junctions, integrins, and cadherins, enabling the exchange of ions, metabolites, and signaling molecules between adjacent cells [116]. This type of communication is vital for angiogenesis, as it can initiate signaling cascades that regulate endothelial cell migration, proliferation, and differentiation—key steps in the formation of new blood vessels. Additionally, cells in co-culture systems release various soluble compounds, such as growth factors (e.g., VEGF and FGF), cytokines, and chemokines [117]. These soluble factors can be taken up by surrounding cells, influencing their behavior.

### 4.2. Indirect (Trans-Well) Co-Culture Systems

Indirect co-culture models involve the physical disassociation of different cell types using membrane inserts, trans-well chambers, or micro-patterned setups. These models are particularly useful for analyzing cell–cell interactions under normal conditions, as well as specific environmental stresses, such as high glucose or hypoxia, and during differentiation processes [118].

In indirect co-culture systems, secreted factors can be examined by using inserts with a trans-well porous membrane, which allows for the cultivation of two distinct populations of cells. Cells cultured in the trans-well insert can be further co-cultured in a dish with another cell type, facilitating the study of cellular communication without direct physical contact. This setup specifically focuses on paracrine signaling, where the secretion of signaling molecules from one cell type affects the behavior of another, highlighting the significance of trophic factor secretion in cell differentiation [119].

The advantages of indirect co-culture models include the ability to identify population-specific cellular changes, facilitate bidirectional signaling, and maintain cell polarity [120]. By utilizing inserts that separate the cell populations with a permeable membrane, researchers can effectively control cell–cell interactions while allowing for the unimpeded flow of secreted factors (Figure 2) [121]. This unique characteristic of indirect co-culture models makes them valuable tools for investigating paracrine-only interactions, as communication between the cells occurs solely through their secretory factors (Table 2) [122].

### 4.3. 3D Co-Culture Systems

**Figure 3:**
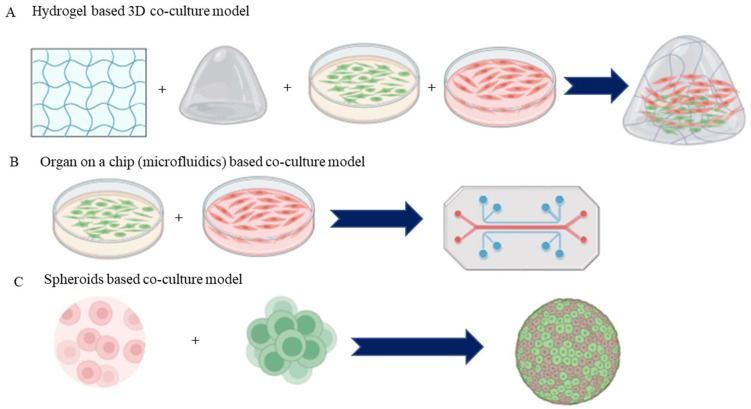
Advanced co-culture systems used in vascular disease. (**A**) Scaffold (hydrogel)-based co-culture models mimic the extracellular matrix and are used to study cell migration and proliferation. (**B**) Microfluidic co-culture system encompasses a dynamic culture environment (shear stress of the blood flow) to the co-cultured cells to study the cell physiology and drug testing. (**C**) Heterotypic 3D spheroids consist of multiple cell types that can mimic in vivo physiology.

Three-dimensional (3D) co-culture systems have emerged as a powerful alternative to traditional two-dimensional (2D) cultures, addressing the limitations of classic models by restoring in vivo conditions within multicellular microtissues (MTs). These systems can incorporate natural or synthetic biomaterials, known as scaffolds, to enhance the physiological relevance of the cultures (Table 2). Research has shown that only 3D technologies utilizing co-cultures can effectively mimic key aspects of cellular heterogeneity and microenvironmental factors essential for tumor growth and vascular studies [136,137]. In the cardiovascular field, existing 3D models primarily fall into two categories: those utilizing a scaffold matrix—typically a hydrogel (Figure 3)—that supports a contracting MT, known as engineered heart tissue (EHT), and those forming smaller cellular aggregates (spheroids) through self-assembly without scaffold proteins [138,139]. Growing literature highlights the utilization of scaffold-free spheroids in the area of drug testing, often involving co-cultures of various cell types, including rodent or human primary stem cells, cardiomyocytes, fibroblasts, and endothelial cells. The integration of advanced technologies, such as microfluidics and micro physiological platforms, further enhances the functionality of these models by facilitating nutrient flow and cellular interaction [140,141]. Although larger tissue formats like multi-layered cell sheets are primarily developed for regenerative medicine due to their complexity and cost, the term “organoid” is increasingly used, albeit with caution, as it traditionally refers to self-organizing structures formed by stem cells. While some degree of self-organization has been observed in vascular networks, the replication of fully functional vascularized organs with a pumping mechanism remains unachievable in vitro [142,143]. Overall, 3D co-culture systems allow for enhanced cell-to-cell communication and better mimicry of in vivo tissue architecture, promoting complex behaviors such as migration and differentiation [144]. These systems are particularly valuable for studying interactions among different cell types, such as endothelial and smooth muscle cells, thereby providing insights into processes like angiogenesis and vascular remodeling [145,146]. Furthermore, 3D co-culture models have proven invaluable in drug discovery, as they more accurately predict cellular responses to therapeutic agents, offering improved assessments of drug pharmacology.

### 4.4. Organ-on-a-Chip Co-Culture Systems

Organ-on-a-chip (OoC) technology depicts a cutting-edge development in biomedical research, providing an innovative platform for modelling human organ functions and studying cellular interactions within a controlled microenvironment. These systems integrate living cells into microfluidic devices, mimicking the physiological conditions of specific organs while allowing for the study of complex cellular behaviors and interactions [147,148].

In OoC co-culture systems, multiple cell types, such as endothelial cells, stromal cells, and epithelial cells, are strategically placed within separate, but interconnected microchannels. This design enables the simulation of tissue architecture and the dynamic interplay between different cell populations, closely resembling the in vivo tissue environment (Figure 3) [149]. By facilitating cell-to-cell communication through paracrine signaling and mechanical interactions, organ-on-a-chip systems provide valuable insights into the pathophysiology of various diseases, including vascular disorders [150]. A key advantage of organ-on-a-chip technology is the propensity to recreate the microenvironmental conditions necessary for studying disease processes. For instance, by adjusting the flow rates and biochemical gradients within the chip, researchers can replicate physiological conditions such as shear stress, nutrient supply, and oxygen levels [151,152]. This level of control allows for the investigation of how these factors influence cellular interactions and responses, providing a more comprehensive understanding of vascular diseases [153].

Furthermore, organ-on-a-chip methods have been instrumental in drug testing and development. By incorporating human primary cells and relevant extracellular matrix components, these systems enable researchers to assess drug efficacy and toxicity in a more relevant context than traditional 2D cultures and animal models [154]. This approach helps to narrow the gap between laboratory findings and clinical outcomes, facilitating the development of more effective therapeutic strategies.

### 4.5. Spheroid and Organoid Co-Culture Systems

Spheroid and organoid co-culture systems are advanced in vitro models that replicate the three-dimensional architecture and functionality of tissues, allowing for a near precise representation of cellular interactions and behaviors compared to traditional two-dimensional cultures.

#### 4.5.1. Spheroid Co-Culture Systems

Spheroids are three-dimensional aggregates of cells that can be formed from various cell types, including cancer cells, stem cells, and primary cells. These cellular clusters provide a more physiologically relevant environment for studying cellular interactions, signaling pathways, and drug responses. In co-culture setups, spheroids composed of different cell types can be engineered to investigate the dynamics of cell–cell interactions, such as those between tumor cells and stromal or immune cells (Figure 3) [155].

Spheroid co-cultures enable researchers to explore the roles of various microenvironmental factors, including extracellular matrix components, oxygen gradients, and nutrient availability, in influencing cellular behavior and function [156]. For example, co-culturing tumor spheroids with immune cells allows for the examination of immune responses to cancer and the effects of therapeutic agents in a setting that closely mimics in vivo conditions [157].

#### 4.5.2. Organoid Co-Culture Systems

Organoids are miniature, self-organizing 3D structures acquired from cells like stem cells and progenitor cells that can differentiate into specific organ-like tissues. These systems closely resemble the architecture and functionality of actual organs, making them powerful tools for studying organ-specific diseases and drug responses [158,159]. Organoids can be derived from various tissues, including the intestine, liver, pancreas, and brain [160,161].

In co-culture systems, organoids can be combined with different cell types, such as endothelial cells, fibroblasts, and immune cells, to create more intricate tissue models. This co-culture approach allows researchers to investigate how different cell types interact within the organoid microenvironment and how these interactions impact tissue function, regeneration, and disease progression. Co-culturing intestinal organoids with immune cells can provide insights into gut immunology and inflammatory bowel diseases [162].

### 4.6. Microcarrier-Based Co-Culture Systems

Microcarrier-based co-culture systems utilize small biocompatible beads or particles to facilitate the growth and interaction of various cell types in a three-dimensional environment, making them particularly advantageous for large-scale cell culture applications. These systems enable researchers to investigate the interactions between different cell populations, such as endothelial and smooth muscle cells, which are crucial for understanding the cellular mechanisms underlying vascular disease and repair [163]. The direct contact allowed by microcarriers promotes essential cell–cell interactions, including paracrine and juxtacrine signaling, which are critical for mimicking in vivo conditions [164]. Additionally, microcarriers enhance scalability and versatility in research, as they can be functionalized with specific extracellular matrix proteins to create tailored environments [165]. Their ease of manipulation and reduced dependency on traditional two-dimensional cultures further enhance their relevance in tissue engineering and pharmacological drug screening, ultimately advancing the study of vascular diseases and potential therapeutic applications.

## 5. Applications of Co-Culture Systems in Drug Testing and Therapeutic Development: Screening Potential Therapeutics

Co-culture systems have emerged as powerful tools for screening potential therapeutics targeting specific cellular interactions in vascular diseases. By recreating the complex cellular microenvironments found in vivo, these systems allow researchers to investigate the efficacy and safety of various drug candidates in a more physiologically relevant context than traditional two-dimensional cultures [93].

A primary application of co-culture models in drug screening involves the targeting of interactions between ECs and SMCs. These interactions play a pivotal role in regulating vascular tone, maintaining tissue homeostasis, and contributing to pathological conditions such as atherosclerosis and restenosis. For instance, the communication between ECs and SMCs is crucial for modulating vascular smooth muscle contraction and relaxation, which directly impacts blood pressure and overall vascular health [39,40]. In co-culture systems designed to study EC–SMC interactions, researchers can introduce specific inhibitors targeting signaling pathways involved in their communication. For example, small molecule inhibitors that block endothelial-derived factors, such as nitric oxide and prostaglandins, can be tested to assess their effects on smooth muscle cell proliferation and migration [41]. By measuring changes in SMC behavior in response to these inhibitors, researchers can gain insights into the therapeutic potential of disrupting aberrant EC–SMC signaling in vascular diseases.

Additionally, co-culture systems allow for the evaluation of drug candidates that enhance beneficial EC–SMC interactions. For example, therapeutic agents that promote the release of protective factors from ECs, such as vascular endothelial growth factor (VEGF) and heme oxygenase-1 (HO-1), can be screened for their ability to improve vascular function and inhibit smooth muscle cell activation [123]. By assessing the outcomes of these treatments within the co-culture environment, researchers can better predict how these therapeutics may perform in vivo. Moreover, co-culture systems facilitate high-throughput screening approaches, enabling the simultaneous evaluation of multiple drug candidates and concentrations. This efficiency accelerates the drug discovery process, allowing researchers to quickly identify promising compounds that specifically target dysfunctional cellular interactions in vascular diseases [166,167].

### 5.1. Developing Targeted Therapies

Insights gleaned from co-culture systems are significantly advancing the development of more precise and targeted therapies for vascular diseases. By providing a detailed understanding of the complex cellular interactions that underpin vascular pathology, these systems enable researchers to identify specific therapeutic targets and develop interventions tailored to modulate these interactions effectively [166].

One key area where co-culture systems have been instrumental is in elucidating the mechanisms of endothelial and smooth muscle cell (SMC) interactions in conditions like atherosclerosis and hypertension. By studying these interactions in a controlled environment, researchers can pinpoint the signaling pathways and molecules involved in vascular remodeling, inflammation, and tone regulation. For example, findings from co-culture studies have revealed the critical role of endothelial-derived factors, such as nitric oxide and endothelin-1, in modulating SMC behavior [41]. Armed with this knowledge, scientists can design targeted therapies that enhance protective endothelial functions or inhibit harmful SMC activation.

Additionally, co-culture systems that incorporate immune cells, like T-cells and macrophages, allow for a deeper exploration of the immune responses contributing to vascular diseases. These systems reveal how immune cell recruitment and activation can influence endothelial dysfunction and promote plaque formation. Consequently, they provide a platform for developing therapies that aim to modulate immune responses in the vascular niche [168]. For instance, drugs that specifically target inflammatory pathways activated by immune cells in the co-culture environment can be explored for their potential to mitigate vascular inflammation and improve outcomes in conditions like atherosclerosis. Furthermore, the use of co-culture systems facilitates the screening of combination therapies, which may offer enhanced efficacy through synergistic effects. By examining how different cell types interact under therapeutic interventions, researchers can identify the most effective combinations of drugs that target multiple facets of vascular disease. This holistic approach not only improves therapeutic outcomes, but also minimizes the risk of adverse effects associated with monotherapy.

The development of targeted therapies is further supported by advances in biomaterials and drug delivery systems that can be tested in co-culture settings. These innovations allow for the localized and sustained delivery of therapeutic agents to specific cell types, improving drug efficacy while reducing systemic side effects. For example, biodegradable scaffolds embedded with anti-inflammatory or pro-angiogenic factors can be utilized in co-culture models to assess their impact on vascular tissue regeneration [169].

### 5.2. Modeling the Effects of Existing Drugs

Co-culture models play a significant role in evaluating the efficacy of existing vascular drugs like statins and anti-inflammatories within a more physiologically relevant setting. By mimicking the complex interactions between various cell types in the vascular system, these models provide a platform to assess how these medications influence cellular behavior and communication in real-time [170].

For instance, when studying statins—widely used for lowering cholesterol and reducing cardiovascular risk—co-culture systems allow researchers to examine their effects, with endothelial cells, smooth muscle cells, and immune cells present in an integrated environment. Through co-culturing these cell types, researchers can evaluate how statins modulate key pathways involved in atherosclerosis, such as reducing oxidative stress and inflammation. This holistic approach enables the assessment of statins’ effects on endothelial function, smooth muscle proliferation, and the immune response, providing a more comprehensive understanding of their therapeutic potential and safety [170].

Similarly, co-culture models are utilized to explore the impact of anti-inflammatory drugs on vascular health. By incorporating immune cells alongside endothelial and smooth muscle cells, researchers can study how these medications influence inflammatory cytokine release and cell signaling pathways in a realistic environment. This is particularly important for drugs aimed at reducing vascular inflammation, as the interactions between endothelial cells and immune cells are critical in the pathogenesis of various vascular diseases [50]. Co-culture studies can reveal whether anti-inflammatory drugs effectively dampen the inflammatory response, thereby preventing endothelial dysfunction and subsequent vascular complications. Moreover, co-culture systems enable the examination of drug interactions and potential synergistic effects among multiple therapies. For example, researchers can assess how combining statins with anti-inflammatory agents impacts cellular responses in a co-culture environment. This approach not only provides insights into the additive or synergistic benefits of combination therapies, but also helps to identify optimal dosing regimens and treatment strategies [170].

### 5.3. Investigating Cell Type-Specific Gene Expression In Vivo

Investigating individual cell type-specific gene expression in vivo and identifying the factors responsible for specific phenotypes is indeed feasible, although it poses challenges due to the complexity of the in vivo environment. Advances in single-cell RNA sequencing (scRNA-seq) allow for the analysis of gene expression profiles at the single-cell level, enabling researchers to isolate specific cell populations within tissues and assess their responses to growth factors and cytokines [171]. Techniques such as RNA in situ hybridization and cell type-specific reporter systems facilitate the identification of gene expression in native tissue environments. Furthermore, conditional knockout models enable the manipulation of specific signaling pathways or growth factors in a cell type-specific manner, allowing for targeted investigations of their roles [172]. Complementing these approaches, proteomic and metabolomic analyses provide insights into how varying levels of cytokines and growth factors influence the functionality and differentiation of individual cell types [173]. Together, these advanced molecular techniques enable the precise examination of gene expression and the identification of key factors that influence cellular phenotypes in vivo.

## 6. Limitations and Challenges of Co-Culture Systems

While co-culture models offer numerous advantages for analyzing cellular interactions and vascular diseases, they also present several limitations and challenges that researchers must navigate.

One major limitation is the complexity of the cellular interactions that can occur within co-culture systems. While these models can mimic certain aspects of the in vivo environment, they may not fully replicate the intricate signaling networks and microenvironmental conditions present in actual tissues [174]. Variations in cell type ratios, cellular states, and local conditions can lead to inconsistencies in results, making it challenging to interpret findings and compare them across different studies [175]. Additionally, the choice of scaffolding materials and the method of co-culture can significantly influence the outcomes. For instance, different biomaterials can elicit varying cellular responses, which may affect the interpretation of drug effects or cellular behavior. The selection of appropriate cell types and their proportions in co-culture systems is also critical; an incorrect choice may not accurately represent physiological conditions or could lead to misleading conclusions.

Another challenge lies in the limited lifespan and viability of cells in co-culture systems. Cells may exhibit altered phenotypes or lose functionality over time, particularly in prolonged culture settings. This can impact the relevance of the results obtained, especially in studies requiring long-term drug exposure or assessment of chronic conditions [93]. Moreover, co-culture systems often require specialized techniques and equipment, such as microfluidics and advanced imaging methods, to fully exploit their potential (Figure 3) [176]. The need for technical expertise and resources can make these models less accessible for some research groups. Finally, while co-culture systems can elucidate cell-to-cell interactions, they may not effectively account for the effect of systemic factors, like hormones, circulating factors, and mechanical forces, which are crucial in the context of vascular diseases. Thus, the results derived from co-culture systems must be studied and analyzed with caution and complemented by in vivo studies to fully understand the complexities of vascular pathology.

## 7. Future Directions and Emerging Trends

Future directions for co-culture systems in vascular disease research promise significant advancements that will enhance their utility in both understanding disease mechanisms and developing therapeutic strategies. There is a growing trend towards integrating a broader range of cell types, including stem cells and fibroblasts, to create more complex and physiologically relevant models. Innovations in 3D bioprinting and microfluidic technologies will enable the replication of vascular architecture and dynamic blood flow conditions, while real-time imaging will facilitate the observation of cellular interactions in action [147,177]. Utilizing patient-derived cells will improve the relevance of the findings to human conditions, and combining omics technologies with co-culture systems will provide comprehensive insights into molecular interactions. Furthermore, these models will have a prominent role in screening and developing targeted therapies, ultimately contributing to advancements in regenerative medicine, particularly for tissue-engineered vascular grafts. Collectively, these emerging trends will deepen our understanding of vascular diseases and enhance the precision of therapeutic approaches.

## 8. Conclusions

In conclusion, co-culture systems represent a transformative approach to studying cellular interactions in vascular disease, offering a similar and physiologically relevant platform compared to traditionally used 2D cultures. By mimicking the complex microenvironments of blood vessels, these systems allow for the exploration of intricate cellular dynamics among various cell types, like endothelial cells, smooth muscle cells, immune cells, and fibroblasts. Insights gained from co-culture studies have illuminated key mechanisms underlying vascular pathologies like hypertension, atherosclerosis, and restenosis. Furthermore, the application of co-culture systems in pharmacological and therapeutic drug development can enhance treatment strategies, enabling targeted interventions that address specific cellular interactions. Despite existing challenges, including technical limitations and variability in experimental setups, ongoing advancements in co-culture methodologies, such as 3D models and microfluidic platforms, promise to expand their applicability and relevance in vascular research. As our understanding of vascular diseases deepens through these innovative approaches, co-culture systems will play a pivotal role in paving the way for novel therapeutic avenues, ultimately improving patient outcomes in cardiovascular health.

## Figures and Tables

**Table 1:** Representation of the signaling pathways altered upon the multicellular interactions under different physiological conditions.

Cellular Interactions	Condition	Signaling Pathways Involved	References
Macrophages and human cardiac microvascular endothelial cells	Hypoxia mediated endothelial dysfunction	Peroxynitrite increased the expressions of hypoxia-inducible factors, (HIF)-1α, HIF-2α, endothelin-converting enzyme (ECE)-1, inducible nitric oxide synthase (iNOS), and cyclooxygenase-2 (COX-2). Reduction in prostacyclin synthase (PGIS)	[49]
Macrophages and endothelial cells	Oxidized low-density, lipoprotein-stimulated atherogenesis	Oxidized LDL-stimulated release of ET-1 from endothelial cells could affect macrophages. Increased expressions of iNOS, COX-2, IL-6, and TNF-α, and decreased expression of Arg-1, mannose receptor C type 1, and IL-10 were found.	[50]
Endothelial cells, macrophages, and pericytes	Vascular angiogenesis	Notch and Jagged signaling	[51]
Monocytes, aortic vascular smooth muscle cells, and endothelial cells	Hyperglycemia-induced arteriosclerosis	Changes in cytokines, interleukin-6 (IL-6), and monocyte chemoattractant protein-1 (MCP-1) expression	[52]
Mouse cardiac endothelial cells and cardiomyocytes from GK rats	Diabetic vascular disease	Exosomes from the myocytes increased the levels of miR-320. Reduced levels of miR-126	[53]
Endothelial cells and cardiomyocytes	Hypoxia-reoxygenation injury	Curcumin treatment inhibited apoptosis and autophagy of cardiomyocytes. Increased the FGF2 levels	[54]
Endothelial cells overexpressing rhSLPI and cardiomyocytes	Hypoxia-reoxygenation injury	Reduced reactive oxygen species production and Bax/Bcl-2, caspase-3, and caspase-8. Activation of p38MAPK and Akt signaling	[55]
Cardiomyocytes and endothelial cells	Ischemia reperfusion injury	Increased NO production Reduction of LDH activity	[56]
Endothelial cells and smooth muscle cells exposed to laminar pulsatile and disturbed flow	Intimal hyperplasia	Defective endothelial monolayer Incorporation of fibronectin in smooth muscle cells	[45]
Endothelial cells and smooth muscle cells in a fibrin gel scaffold, with addition of monocytes later	Atherosclerosis	Infiltration of lipids into macrophages Development of foam cell was studied.	[47]
Endothelial cells, smooth muscle cells, and macrophages	Atherosclerosis	Increase in the expression of IL6, IL8, CXCL1/GROα, and CCL2/MCP1 Elevation of inflammatory pathways such as JAK/STAT, NFκB, and Jun signaling	[48]

**Table 2:** Overview of the various types of co-culture approaches employed to investigate cardiovascular diseases.

Type of Co-Culture	Description	Advantages	Limitations	References
Direct co-culture	Various cell types are seeded in the same culture dish, which allows cell-to-cell communication via gap junction, adherens, and paracrine signaling.	Able to analyze contact-based and non-contact-based cellular interactions. Simple, easy, and cost-effective way to culture.	Difficult to achieve equal amounts of cellular densities of both the cells studied. One cell type could grow fast/slow and might not mimic the exact vascular environment. Culture media used should be adaptable for both the cell types used.	[123,124]
Direct co-culture with trans-well	Different cell types are seeded on the upper and lower sides of the porous trans-well inserts.	Direct cell–cell contact allows for study of the physical contact interactions between the cell types. Can demonstrate cell adhesion, permeability, and migration towards the other cell type. Different culture media can be used for the different cell populations across the trans-well.	Trans-well membranes are expensive, and they cannot be reused. There is no difference whether the pathological condition developed is based on contact-dependent or contact-independent signals.	[125,126]
Indirect co-culture, trans-well based	Two cell types are cultured in different chambers of the trans-well membrane, and the distance between them allows communication only through soluble factors in the culture media.	Can be used to study cell–cell interactions, drug permeability, and drug transport.	Cellular communication restricts to soluble secretions (growth factors, cytokines, and extracellular vesicles) alone and lacks signaling through physical contact to mimic in vivo environment. Expensive.	[98,127,128]
Conditioned media-based indirect co-culture	Cell secretions of one cell type (conditioned media) when transferred to another cell type, which can modulate the cell behavior.	Easy to establish and provides secretory factors to modulate the other cell type. Conditioned media can be frozen and can be used on other cell type later.	Unidirectional. It is used to study only secretory factor-based signaling and lacks contact signaling.	[129,130]
3D co-culture, scaffold based	Encapsulating different cell types in a 3D scaffold, which can provide topography and mechanical stimulus needed reflecting physiological microenvironment.	Mimics more of an in vivo condition and allows for the study of cell morphology, behavior, function, cell–cell contact signaling, and paracrine signaling. Recapitulates the vascular microenvironment realistically. Multiple cellular interactions (both physical and secretory) are feasible.	Expensive. Needs more time to optimize the culture. Proteolytic separation of a single layer of cells is difficult. Repeatability of the experiments is difficult.	[131,132]
Microfluidics-based co-culture	Dynamic fluid manipulation system designed for micrometer sized channels. It mimics physiological microenvironment, which can culture multiple cell types.	Regulation of signal gradients and perform simulation of physiological microenvironment such as shear stress. Reliable platform for drug screening and vascular modeling.	Needs external devices like pumps, connectors, and valves to function. Difficult to optimize and repeat the experiments. Expensive.	[133]
Organoids-based co-culture	Self-organizing 3D cellular structures that can recapitulate organs (cardiac organoid–endothelial cells, cardiomyocytes, fibroblasts, etc.).	Modeling cardiogenesis, drug screening, and testing and also in tissue engineering.	Difficult to optimize. Hard to reproduce. Less or insufficient vascularization limits the applicability of cardiac organoid.	[134,135]

## Data Availability

Not applicable.
